# A Multicenter, International Cohort Analysis of 1435 Cases to Support Clinical Trial Design in Acute Pancreatitis

**DOI:** 10.3389/fphys.2019.01092

**Published:** 2019-09-04

**Authors:** Nelli Farkas, Lilla Hanák, Alexandra Mikó, Judit Bajor, Patrícia Sarlós, József Czimmer, Áron Vincze, Szilárd Gódi, Dániel Pécsi, Péter Varjú, Katalin Márta, Péter Jenő Hegyi, Bálint Erőss, Zsolt Szakács, Tamás Takács, László Czakó, Balázs Németh, Dóra Illés, Balázs Kui, Erika Darvasi, Ferenc Izbéki, Adrienn Halász, Veronika Dunás-Varga, László Gajdán, József Hamvas, Mária Papp, Ildikó Földi, Krisztina Eszter Fehér, Márta Varga, Klára Csefkó, Imola Török, Farkas Hunor-Pál, Artautas Mickevicius, Elena Ramirez Maldonado, Ville Sallinen, János Novák, Ali Tüzün Ince, Shamil Galeev, Barnabás Bod, János Sümegi, Petr Pencik, Attila Szepes, Andrea Szentesi, Andrea Párniczky, Péter Hegyi

**Affiliations:** ^1^Institute of Bioanalysis, Medical School, University of Pécs, Pécs, Hungary; ^2^Institute for Translational Medicine, Medical School, University of Pécs, Pécs, Hungary; ^3^Department of Gastroenterology, First Department of Medicine, Medical School, University of Pécs, Pécs, Hungary; ^4^First Department of Medicine, University of Szeged, Szeged, Hungary; ^5^Szent György University Teaching Hospital, Fejér County, Székesfehérvár, Hungary; ^6^Bajcsy-Zsilinszky Hospital, Budapest, Hungary; ^7^Department of Internal Medicine, Division of Gastroenterology, Faculty of Medicine, University of Debrecen, Debrecen, Hungary; ^8^Dr. Réthy Pál Hospital, Békéscsaba, Hungary; ^9^County Emergency Clinical Hospital, University of Medicine, Pharmacy, Sciences and Technology of Targu Mures, Targu Mures, Romania; ^10^Vilnius University Hospital Santariskiu Klinikos, Vilnius, Lithuania; ^11^Consorci Sanitari del Garraf, Barcelona, Spain; ^12^Department of Transplantation and Liver Surgery, Helsinki University Central Hospital, University of Helsinki, Helsinki, Finland; ^13^Pándy Kálmán Hospital of County Békés, Gyula, Hungary; ^14^School of Medicine, Hospital of Bezmialem Vakif University, Istanbul, Turkey; ^15^Saint Luke’s Clinical Hospital, St. Petersburg, Russia; ^16^Dr. Bugyi István Hospital, Szentes, Hungary; ^17^Borsod-Abaúj-Zemplén County Hospital, University Teaching Hospital, Miskolc, Hungary; ^18^Centrum Péče o Zažívací Trakt, Vítkovická Nemocnice a.s., Ostrava, Czechia; ^19^Department of Gastroenterology, Bács-Kiskun County Hospital, Kecskemét, Hungary; ^20^Heim Pál National Institute of Pediatrics, Budapest, Hungary; ^21^Clinical Medicine Doctoral School, University of Szeged, Szeged, Hungary; ^22^Momentum Gastroenterology Multidisciplinary Research Group, Hungarian Academy of Sciences, University of Szeged, Szeged, Hungary

**Keywords:** acute pancreatitis, C-reactive protein, white blood cell, trial design, sample size calculation

## Abstract

**Background:**

C-reactive protein level (CRP) and white blood cell count (WBC) have been variably used in clinical trials on acute pancreatitis (AP). We assessed their potential role.

**Methods:**

First, we investigated studies which have used CRP or WBC, to describe their current role in trials on AP. Second, we extracted the data of 1435 episodes of AP from our registry. CRP and WBC on admission, within 24 h from the onset of pain and their highest values were analyzed. Descriptive statistical tools as Kruskal–Wallis, Mann–Whitney *U*, Levene’s *F* tests, Receiver Operating Characteristic (ROC) curve analysis and AUC (Area Under the Curve) with 95% confidence interval (CI) were performed.

**Results:**

Our literature review showed extreme variability of CRP used as an inclusion criterion or as a primary outcome or both in past and current trials on AP. In our cohort, CRP levels on admission poorly predicted mortality and severe cases of AP; AUC: 0.669 (CI:0.569–0.770); AUC:0.681 (CI: 0.601–0.761), respectively. CRP levels measured within 24 h from the onset of pain failed to predict mortality or severity; AUC: 0.741 (CI:0.627–0.854); AUC:0.690 (CI:0.586–0.793), respectively. The highest CRP during hospitalization had equally poor predictive accuracy for mortality and severity AUC:0.656 (CI:0.544–0.768); AUC:0.705 (CI:0.640–0.769) respectively. CRP within 24 h from the onset of pain used as an inclusion criterion markedly increased the combined event rate of mortality and severe AP (13% for CRP > 25 mg/l and 28% for CRP > 200 mg/l).

**Conclusion:**

CRP within 24 h from the onset of pain as an inclusion criterion elevates event rates and reduces the number of patients required in trials on AP.

## Introduction

Acute pancreatitis (AP) is one of the most common acute gastrointestinal pathology needing hospitalization in the United States (Peery et al., 2012). It is often mild, but in moderate and severe cases it can have a mortality of around 30% despite current treatment options (Gompertz et al., 2013; Parniczky et al., 2016). In our previous cohort analysis on the first 600 subjects recorded in our multicentric, and multinational AP database severe disease developed in 8.8% of all cases (Parniczky et al., 2016).

There are few clinical trials on AP despite its burden (Szentesi et al., 2016). There were 183 registered clinical studies on ClinicalTrials.gov in November 2018. This is in the context of 1914 studies on IBD, 395 on Helicobacter pylori and 235 on colonic polyps registered at the same time.

High-quality clinical trials on AP are difficult to conduct as the most important clinical outcomes, such as severe acute pancreatitis (SAP) and death, have low event rates (Gompertz et al., 2013; Parniczky et al., 2016). Furthermore, at the time of the diagnosis of AP, the disease course and outcome are difficult to predict (Cho et al., 2015). As a result, there are many trials on AP that have been designed with primary outcomes other than mortality and SAP.

The most obvious and simplest markers of inflammation are C-reactive protein level (CRP) and white blood cell count (WBC). They are widely available and readily reported in nearly any healthcare settings. Therefore, trials on AP have been using inflammatory markers as inclusion criteria and as primary outcomes too. However, there is no currently available evidence-based information and practical guideline on their use.

Our aim was to assess the past and current role of CRP and WBC in clinical trials on AP and to provide evidence from a high-quality large multinational cohort analysis to guide clinical researchers on the most appropriate role of CRP and WBC in future clinical trials.

## Materials and Methods

### The Two Main Domains

First, we reviewed the studies on AP that have used CRP or WBC as inclusion criterion or as an outcome to understand their past and current role.

Second, we analyzed data from a large prospectively collected cohort of AP patients to assess the potential future role of CRP and WBC in the design of trials.

### Literature Review

We searched PubMed on January 9, 2019 for the query: random^∗^ AND “acute pancreatitis.” The records were managed by EndNote X7.4, software (Clarivate Analytics, Philadelphia, PA, United States). Studies published before January 1, 2000 were excluded. The records were screened by title, by abstract and by full text.

In addition, we searched the study registries ClinicalTrials.gov and International Standard Randomised Controlled Trial Number (ISRCTN) by using the query “acute pancreatitis” on 15/01/2019 for ongoing studies on AP.

Studies from both searches were selected that used CRP or WBC as an inclusion criterion or primary outcome in trials on AP.

The aggregated yield of both search strategies was screened and data were extracted on the role, timing, and thresholds of CRP and WBC.

### Cohort Analysis

#### The Setting of the Study and the Database

Subjects with AP were enrolled in our large multicentric and multinational registry on AP hospitalization from 13 countries and 29 centers (Supplementary Figure 1). Their detailed data were uploaded to the database for the hospitalization of AP. The project was approved by the Scientific and Research Ethics Committee of the Medical Research Council on August 15, 2012 (22254-1/2012/EKU), also by the participating centers’ ethical boards and subjects in the study provided informed and written consent. The study protocol conforms to the ethical guidelines of the 1975 Declaration of Helsinki as reflected in *a priori* approval by the institution’s human research committee. Information on the quality of data is provided in the Supplementary Table 1.

### Definition of Acute Pancreatitis

Acute pancreatitis was defined as the presence of at least two of the following criteria: abdominal pain, at least threefold rise in the pancreatic enzymes and evidence of pancreatitis on imaging (Banks et al., 2013). Severity grades were defined by the modified Atlanta criteria (Banks et al., 2013).

### Measurement of CRP and WBC

This database includes data from multiple centers and all contributing centers complied with all the necessary laboratory requirements and guidelines. CRP and WBC were uploaded onto the prospective database in units of mg/l and G/l, respectively. If local measurements used different units then the results were calculated and recorded in the above units.

### Data Extraction

We extracted data on the onset of pain, CRP, WBC, demographics (age and gender) and etiology, severity, and mortality of AP. Data extraction included patients enrolled between August 2012 and September 2017.

### Statistical Analysis

Prior to the analysis of the dataset, descriptive statistical tools were used to describe the basic characteristics. To observe differences between the severity groups, we applied Kruskal–Wallis test with Mann–Whitney *U*-test as *post hoc*, because of the non-parametric behavior of the variables. The beginning of pain before admission influences the levels of the inflammatory parameters, therefor the CRP and WBC levels on admission were adjusted for the duration of pain. For verification of this statement, the homoscedasticity of the original and corrected on admission CRP and WBC were examined with Levene’s *F* test. Receiver Operating Characteristic (ROC) curve analysis and AUC (Area Under the Curve) with 95% confidence interval (CI) determination were used to check the ability of CRP and WBC for classification of severity and mortality. According to the value of AUC, the accuracy of the test can be classified as followed: 0.5–0.6 fail, 0.6–0.7 poor, 0.7–0.8 fair, 0.8–0.9 good and above 0.9 excellent. All analyses were conducted using IBM-SPSS Statistical Software version 25 (IBM Corporation, Armonk, NY, United States).

## Results

### Role of CRP and WBC in Past and Ongoing Clinical Studies

Our search criteria yielded a total of 2021 records. After the exclusion of studies from before 2000, there were 1664 left. After screening by title 136 eligible abstracts remained and 107 full-text articles were reviewed. This strategy identified 17 studies where CRP was an inclusion criterion, 10 studies where CRP was a primary outcome (Powell et al., 2000; Louie et al., 2004; Pearce et al., 2006; Guo et al., 2013; Sun et al., 2013; Cui et al., 2014; Vege et al., 2015; Choosakul et al., 2018; de-Madaria et al., 2018; Jin et al., 2018) and two studies where CRP was both an inclusion criterion and primary outcome.

We found three studies on ClinicalTrials.gov where CRP is a primary outcome (NCT03686618, NCT02692391, NCT02885441) and two studies where CRP is an inclusion criterion (NCT00894907, NCT03082469). A further study was registered both on ClinicalTrials.gov and ISRCTN which uses CRP as a primary outcome (NCT03684278, ISRCTN16935761).

Furthermore, CRP was used as a variable inclusion criterion. Twelve studies used a cut off level of 150 mg/l, four studies 120 mg/l, and three studies 100 mg/l (NCT00894907, NCT03082469). One study specified a cut off level at 120 mg/l within 24 h from admission and 200 mg/l within 48 h from admission and another study just mentions elevated CRP. One study required high CRP on admission, two studies within 24, and one study within 48 h from admission. From the onset of symptoms six studies required CRP within 48 (NCT00894907, NCT03082469), four studies within 72, one study within 120 h and six studies did not specify the timeframe. The details and the references are shown in the Supplementary Table 2 and Supplementary References.

White blood cell count is used as an optional inclusion criterion only in two studies with a cut off level of > 16G/l.

### Data Quality, Demographics, and Basic Characteristics of the Cohort

Our analysis included data of 1435 episodes of AP. We had 100% data on age, gender, severity, mortality, and length of hospitalization. WBC and CRP on admission were available in 1288 (89.9%) and 1177 cases (82.0%), respectively. WBC and CRP, within 24 h from the onset of pain, were available in 691 (48.2%) and in 627 patients (43.7%) respectively. There were 79 (5.51%) severe, 371 (25.85%) moderately severe, 985 (68.64%) mild AP, and altogether 34 patients died (2.37%). The details of the descriptive statistics (demographics, etiology) of the cohort are shown in the Supplementary Figure 2. Further details of the data on CRP level and WBC count are shown in Supplementary Figures 4–6.

### On Admission CRP Cannot Predict the Mortality or Severity

**FIGURE 1:**
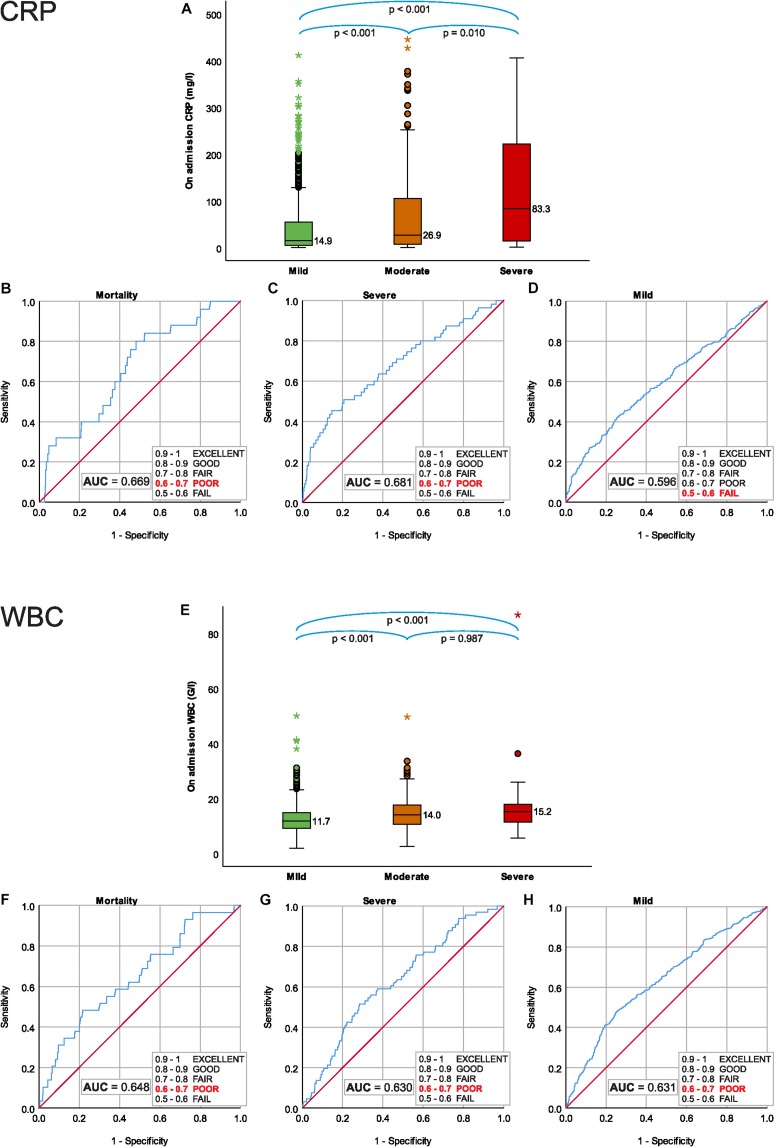
On admission CRP; **(A)** median CRP for severity grades of acute pancreatitis (AP), **(B)** predictive accuracy for mortality of AP, **(C)** predictive accuracy for severe AP, **(D)** predictive accuracy for mild AP. On admission WBC; **(E)** median WBC for severity grades of AP, **(F)** predictive accuracy for mortality of AP **(G)** predictive accuracy for severe AP, and **(H)** predictive accuracy for mild AP.

On admission median CRPs were directly associated with the severity of AP, 14.90 (IQR: 5.00–54.60) mg/l in mild, 26.90 (IQR: 6.93–105.95) mg/l in moderately severe, and 83.30 (IQR: 13.50–222.70) mg/l in severe (mild vs. moderately severe, *p* < 0.001; mild vs. severe, *p* < 0.001; moderately severe vs. severe AP, *p* = 0.01) (Figure 1A).

Receiver Operating Characteristic analyses showed poor predictive accuracy of CRP on admission for mortality and severe AP, AUC: 0.669 (CI: 0.569–0.770) (Figure 1B) and AUC: 0.681 (CI: 0.601–0.761) (Figure 1C) respectively, and it failed to predict mild AP, AUC: 0.596 (CI: 0.559–0.663) (Figure 1D).

### On Admission, WBC Cannot Predict the Mortality or Severity

On admission median WBC counts were significantly lower in mild AP 11.70 (IQR: 9.16–14.80) G/l, than in moderately severe 14.00 (IQR: 10.50–17.54) G/l and 15.24 (IQR: 11.30–17.95) G/l in severe AP (mild vs. moderately severe AP, *p* < 0.001; mild vs. severe AP, *P* < 0.001; moderately severe vs. severe AP, *P* = 0.987) (Figure 1E).

ROC analyses showed poor predictive accuracy of WBC on admission for mortality, severe and mild AP, AUC: 0.648 (CI: 0.546–0750) (Figure 1F) and AUC: 0.630 (CI: 0.563–0.696) (Figure 1G) and AUC: 0.631 (CI: 0.598–0.664) (Figure 1H), respectively.

### The CRP Within 24 h From the Onset of Pain Cannot Predict the Mortality or Severity

**FIGURE 2:**
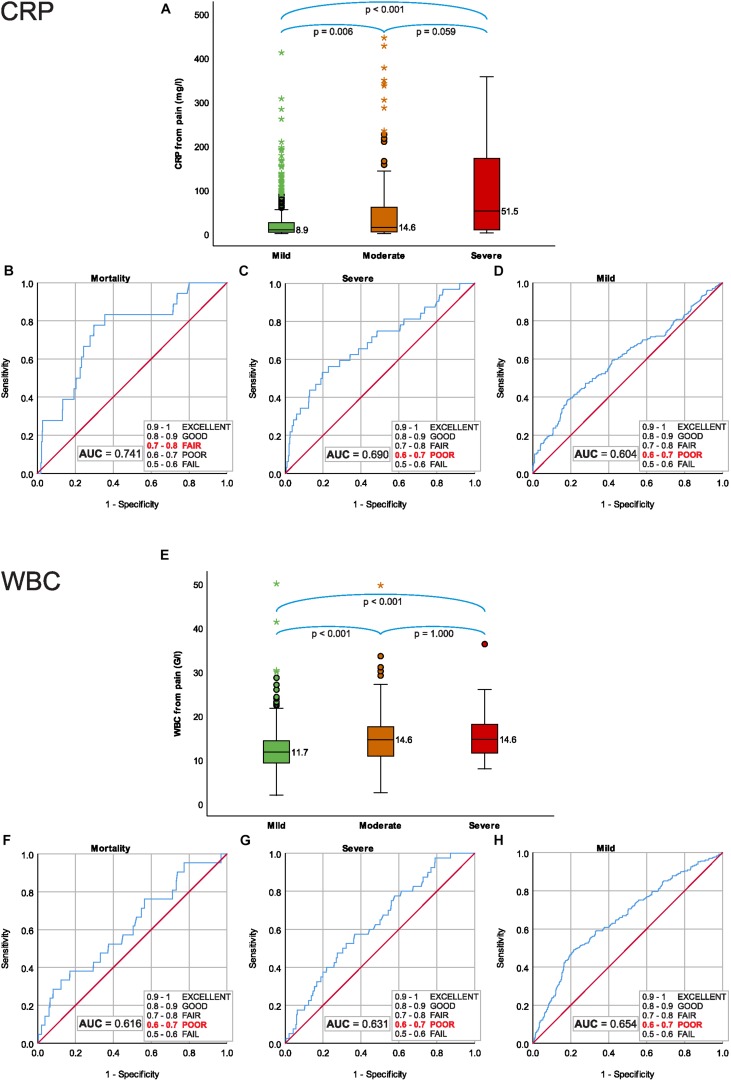
C-reactive protein level within 24 h from the onset of pain; **(A)** median CRP for severity grades of acute pancreatitis (AP), **(B)** predictive accuracy for mortality of AP, **(C)** predictive accuracy for severe AP, **(D)** predictive accuracy for mild AP. WBC within 24 h from the onset of pain; **(E)** median WBC for severity grades of AP, **(F)** predictive accuracy for mortality of AP, **(G)** predictive accuracy for severe AP, and **(H)** predictive accuracy for mild AP.

Median CRP within 24 h from the onset of pain were significantly lower in mild 8.90 (IQR: 3.60–25.10) mg/l and moderately severe 14.60 (IQR: 4.15–60.46) mg/l, than in severe AP 51.45 (IQR: 7.85–175.53) mg/l (mild vs. moderately severe AP: *P* = 0.006; mild vs. severe AP: *P* < 0.001; moderately severe vs. severe AP: *P* = 0.059) (Figure 2A).

Receiver Operating Characteristic analyses showed a fair predictive accuracy of CRP within 24 h for mortality AUC: 0.741 (CI: 0.627–0.854) (Figure 2B) and poor predictive accuracy for both severe and mild AP, AUC: 0.690 (CI: 0.586-0.793) (Figure 2C) and AUC: 0.604 (CI: 0.554-0.653), respectively (Figure 2D).

### WBC Within 24 h From the Onset of Pain Cannot Predict the Mortality or Severity

Median of the WBC within 24 h from the onset of pain was significantly lower in mild 11.70 (IQR: 9.24–14.32) G/l, than in moderately severe 14.55 (IQR: 10.80–17.51) G/l and in severe AP 14.60 (IQR: 11.45–18.07) G/l (mild vs. moderately severe AP, *p* < 0.001; mild vs. severe AP, *P* < 0.001; moderately severe vs. severe AP, *P* = 1.000) (Figure 2E).

ROC analyses showed poor predictive accuracy of WBC within 24 h for mortality, severe and mild AP, AUC: 0.616 (CI: 0.490–0.742) (Figure 2F), AUC: 0.631 (CI: 0.547–0.715) (Figure 2G) and AUC: 0.654 (CI: 0.610–0.698) (Figure 2H), respectively.

### Increasing the CRP Level on Admission as an Inclusion Criterion Elevates the Event Rates of Severe AP and Mortality

Although CRP cannot predict the severity or mortality of AP, higher levels on admission increase the event rate of mortality and severity and their composite. However, a decreasing proportion of AP patients have higher levels of CRP and WBC on admission (Table 1).

**TABLE 1:** The association between on admission C-reactive protein levels (CRP) and white blood cell count (WBC) and the severity and mortality of acute pancreatitis.

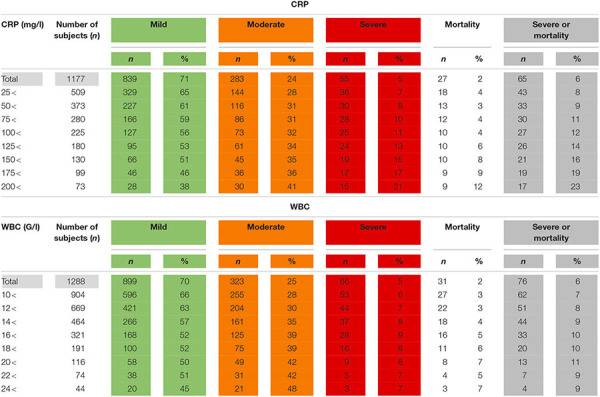

Limiting the on admission CRP and WBC to patients who presented within 24 h from the onset of the pain, the potential number of subjects dropped, but severity and mortality were higher compared to all admissions (Table 2).

**TABLE 2:** The association between C-reactive protein levels (CRP) and white blood cell count (WBC) within 24 h from the onset of pain and the severity and mortality of acute pancreatitis.

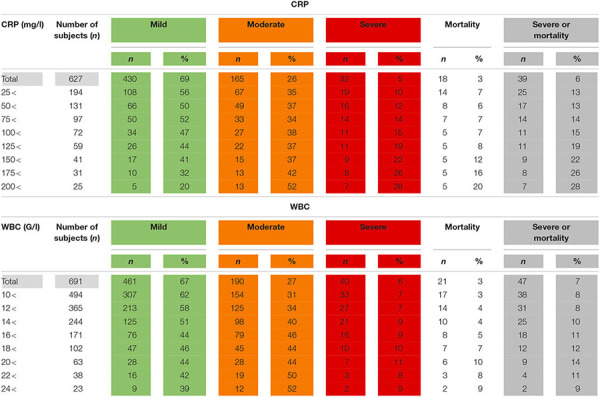

White blood cell count did not show a clear relationship with the outcomes of AP regardless of the timing of the measurement (Tables 1, 2).

**FIGURE 3:**
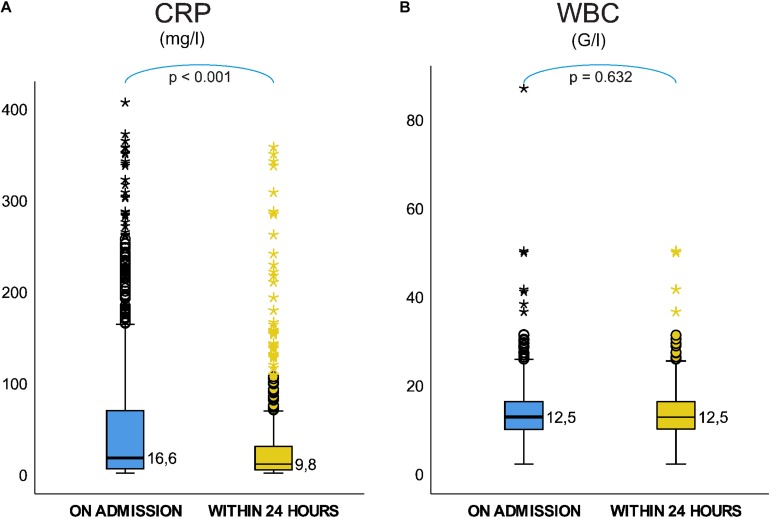
**(A)** Testing of homoscedasticity reveals a significant difference in the dispersion of CRP values limited to the 24 h from the onset of pain, compared to all on admission CRP values’ dispersions. **(B)** There is no difference in the dispersion of values of WBC.

Analysis of the homoscedasticity of CRP and WBC for the duration of pain revealed that there were significant differences between the homoscedasticity of the CRP on day 1, 2, and 4, the details are in Supplementary Figure 3. Limiting the admission CRP to those within 24 h from the onset of pain significantly increased the homoscedasticity *P* < 0.001 (Figure 3A). The same limitation did not lead to any significant change in the homoscedasticity of the WBC, *P* = 0.632 (Figure 3B).

### The Maximum CRP Cannot Predict the Mortality or Severity

**FIGURE 4:**
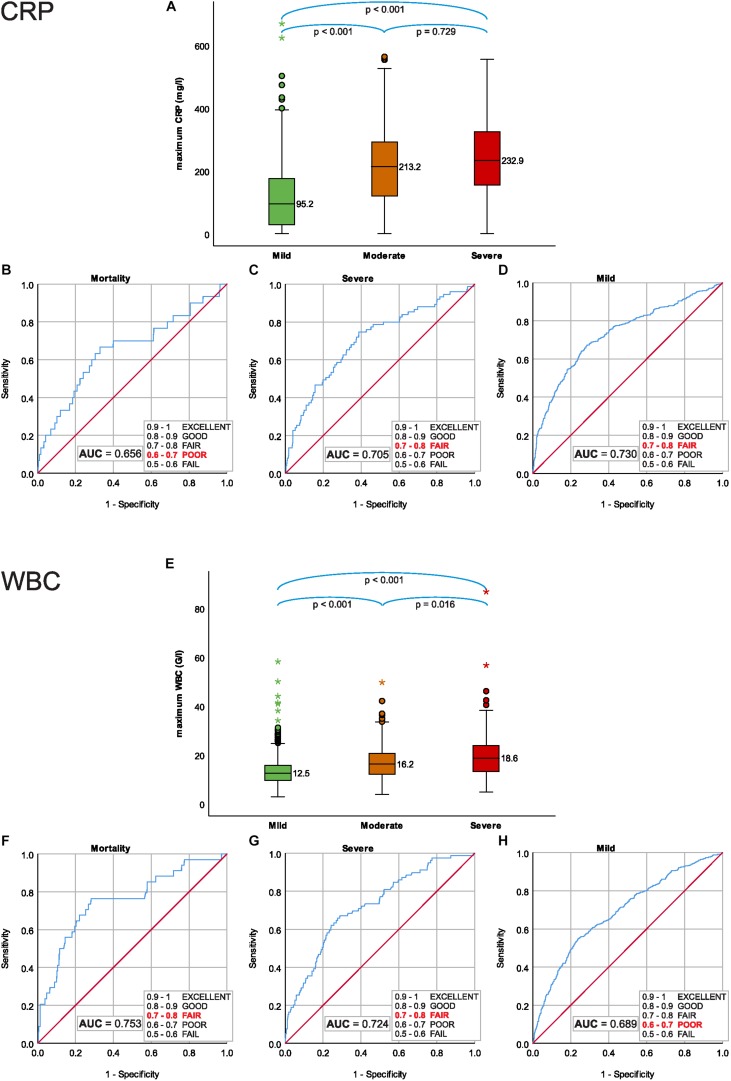
Maximum CRP during hospitalization; **(A)** median CRP for severity grades of acute pancreatitis (AP), **(B)** predictive accuracy for mortality of AP, **(C)** predictive accuracy for severe AP, **(D)** predictive accuracy for mild AP. Maximum WBC during hospitalization; **(E)** median WBC for severity grades of AP, **(F)** predictive accuracy for mortality of AP, **(G)** predictive accuracy for severe AP, and **(H)** predictive accuracy for mild AP.

Median of maximum CRP was significantly lower in mild, 95.18 (IQR: 28.73–175.53) mg/l and in moderately severe 213.20 (IQR: 119.20–292.00) mg/l, than in severe AP 232.90 (IQR: 147.50–333.95) mg/l (mild vs. moderately severe AP, *p* < 0.001; mild vs. severe AP, *P* < 0.001; moderately severe vs. severe AP, *P* = 0.729) (Figure 4A).

Receiver Operating Characteristic analyses showed a poor predictive accuracy of maximum CRP for mortality AUC: 0.656 (CI: 0.544–0.768) (Figure 4B) and a fair predictive accuracy for both severe and mild AP, AUC: 0.705 (CI: 0.640–0.769) (Figure 4C) and AUC: 0.730 (CI: 0.701–0.760), respectively (Figure 4D).

### The Maximum WBC Cannot Predict the Mortality or Severity

Median of maximum WBC was directly associated with the severity of AP, 12.49 (IQR: 9.55–15.68) G/l in mild, 16.16 (IQR: 12.00–20.59) G/l in moderately severe, and 18.63 (IQR: 13.12–24.30) G/l in severe (mild vs. moderately severe AP, *p* < 0.001; mild vs. severe AP, *P* < 0.001; moderately severe vs. severe AP, *P* = 0.016) (Figure 4E).

Receiver Operating Characteristic analyses for maximum WBC showed fair predictive accuracy for mortality and severe AP, AUC: 0.753 (CI: 0.662–0.843) (Figure 4F) and AUC: 0.724 (CI: 0.667–0.782), respectively (Figure 4G) and poor predictive accuracy for mild AP 0.689 (CI: 0.659–0.719) (Figure 4H).

## Discussion

### Diverse Application of CRP in Past and Current Clinical Trials on AP Has Been Lacking High-Quality Evidence

The results of our review proved that CRP is widely used in studies as an inclusion criterion or primary outcome. CRP is more commonly used as an inclusion criterion and its use is highly variable in terms of threshold and timing.

The first study on the role of CRP in the prediction of the outcome of AP was published by Mayer et al. (1984) and concluded that high levels of CRP may predict a severe disease course. Further two studies from the late 1980s drew the same conclusions and pointed out that CRP was higher in AP with severe disease course (Puolakkainen et al., 1987; Wilson et al., 1989).

In most studies where CRP is an inclusion criterion the authors argue that CRP is a predictor of severe disease based on past evidence and cite a consensus article from 1999 by Dervenis et al. (1999), which stated that CRP becomes a good indicator after 48 h. The authors recommended that CRP > 150 mg/l is a marker of severity (Dervenis et al., 1999). The problem with this recommendation is that this consensus is based on data from old, small, and incomplete studies (Gross et al., 1990; Leser et al., 1991; Heath et al., 1993; Pezzilli et al., 1995; de Beaux et al., 1996). At that time, neither the definition nor the classification of AP were the same as they are now and the measurment of CRP was less reliable.

More recently the largest study on the assessment of CRP in AP included 172 patients (Neoptolemos et al., 2000). Other studies reported data from smaller cohorts (Pongprasobchai et al., 2010; Vasudevan et al., 2018).

There is only one study analyzing the role of CRP in AP, since the introduction of revised Atlanta classification, but this is from a tertiary referral center, where patients were admitted within 14 days from the onset of disease and there were only 50 subjects enrolled (Vasudevan et al., 2018). This study calculated a predictive value of CRP with an AUC of 0.8218 for severe AP. However, we must note that these patients were all well into their disease course and we know that CRP peaks after day 3 in AP (Neoptolemos et al., 2000).

In view of the above arguments and the findings of our literature search, it is clear that CRP is widely used, but there is a far too great and unjustified variability of its use both in terms of threshold and its timing.

### Role of CRP in Future Clinical Studies on AP

Based on the results of our analysis, neither CRP nor WBC can predict mortality or severe disease on the day of admission, even if they are restricted to patients who present within less than 24 h from the onset of the pain. The AUC for the predictive value of CRP and WBC regardless their timing did not exceed 0.8, therefore a cut-off cannot be determined and they should not be used to predict severity or mortality.

If we accept that studies should aim to analyze effects of interventions in the early phase of AP as the disease course may more likely to be influenced, then we have to assess the potential role of any inclusion criteria on admission and as soon as possible from the onset of symptoms.

We have shown that CRP limited within 24 h from the onset of pain can increase the homoscedasticity (Figure 3). Therefore, we should adjust this parameter for the onset of pain, that is the duration of the AP.

In order to have the best evidence in the treatment of AP we believe that mortality and severe disease should be the endpoints of high-quality trials.

### Feasibilty

Our data clearly demonstrates that we would be able to reduce the number of participants in the study by increasing the inclusion CRP levels, but we would need to screen an increasing population to find the eligible subjects as less and less subjects would fit the inclusion criterion for enrollment (Tables 1, 2). However, the elevated event rate of severe disease and mortality could reduce the sample size of the study population. In other words, it could compensate clinical researchers for the loss of eligible subjects for enrollment by reaching sufficient number of hard endpoints in smaller sample sizes.

### Sample Size Calculation

To demonstrate the clinical relevance and potential use of our findings, we calculated the sample sizes for a hypothetical study on AP, in which we would like to demonstrate a 50% reduction of the composite endpoint with 80% statistical power and a *P* of 0.05 (Table 3).

**TABLE 3:** Based on the composite endpoints of severe acute pancreatitis (AP) for the CRP levels within 24 h form the onset of pain in our cohort we calculated the sample size for a hypothetical study on AP, in which we would like to demonstrate a 50% reduction of the composite endpoint with 80% statistical power and a *P* of 0.05.

**CRP (mg/l)**	**Number of subjects**		**Severe disease or**		**Estimated sample size in the**	
**within 24 h from**	**in our cohort**		**mortality in our cohort**		**hypothetical study**	
**the onset of pain**			**(composite endpoint)**			
	***n***	**%**	***n***	**%**	**Number of patients to be included in the study**	**Number of patients with AP to be screened**
0<	627	100	39	6	–	–
25<	194	31	25	13	326	1052
50<	131	21	17	13	326	1552
75<	97	15	14	14	300	2000
100<	72	11	11	15	278	2527
125<	59	9	11	19	212	2356
150<	41	7	9	22	178	2543
175<	31	5	8	26	145	2900
200<	25	4	7	28	132	3300

*The table clearly demonstrates that we would be able to reduce the number of participants in the study by increasing the inclusion CRP levels, but we would need to screen an increasing population to find the eligible subjects.*

We would recommend that clinical researchers use CRP within 24 h from the onset of pain as an inclusion criterion. In the design process the researchers should set the threshold of inclusion CRP depending on the practicalities and feasibility of the planned study.

### Inflammatory Markers as Primary Outcome

Our results demonstrate that the highest detected CRP and WBC during the course of AP cannot predict the severity or mortality of the disease (AUC < 0.8) (Figure 4). Therefore, they should not be used arbitrarily, as primary outcomes in trials on AP.

## Conclusion

Here we provide high-quality evidence and a method how to decrease the number of patients required for clinical trials on AP. We demonstrate that by increasing the CRP levels measured within 24 h from the onset of abdominal pain as an inclusion criterion we can elevate the event rates of mortality, and severity. Investigators can set the inclusion CRP level tailored for their study purposes and can test feasibility. Importantly, our data proves that CRP should not be used as a primary outcome. Finally, WBC should not be used neither as an inclusion criterion nor as an outcome in clinical trials on AP.

## Data Availability

The datasets generated for this study are available on request to the corresponding author.

## Ethics Statement

The studies involving human participants were reviewed and approved by Scientific and Research Ethics Committee of the Medical Research Council on August 15, 2012 (22254-1/2012/EKU). The patients/participants provided their written informed consent to participate in this study.

## Author Contributions

NF and PH designed the research and study concept. NF and LH performed the data extraction and analyzed and interpreted the data. NF, LH, BE, and PH wrote the article. ÁV, AS, ED, and PH supervised the study. AlM, JB, PS, JC, ÁV, SzG, DP, PV, KM, PJH, BE, TT, LC, BN, DI, BK, ED, FI, AH, VD-V, LG, JH, MP, IF, KF, MV, KC, IT, FH-P, ArM, EM, VS, JN, AI, ShG, BB, JS, PP, and ASzep enrolled and managed the patients and also conducted a critical revision of the manuscript for important intellectual content. All of the co-authors granted final approval for the manuscript.

## Conflict of Interest Statement

The authors declare that the research was conducted in the absence of any commercial or financial relationships that could be construed as a potential conflict of interest.
