# Supplementary material for: A Multicenter, International Cohort Analysis of 1435 Cases to Support Clinical Trial Design in Acute Pancreatitis
Source: Front Physiol. 2019 Sep 4;10:1092. doi: 10.3389/fphys.2019.01092 (PMC6738025; doi:10.3389/fphys.2019.01092)
Supplement: Supplementary file 1 [file Data_Sheet_1.docx]

**Supplementary Figures and Tables**

**
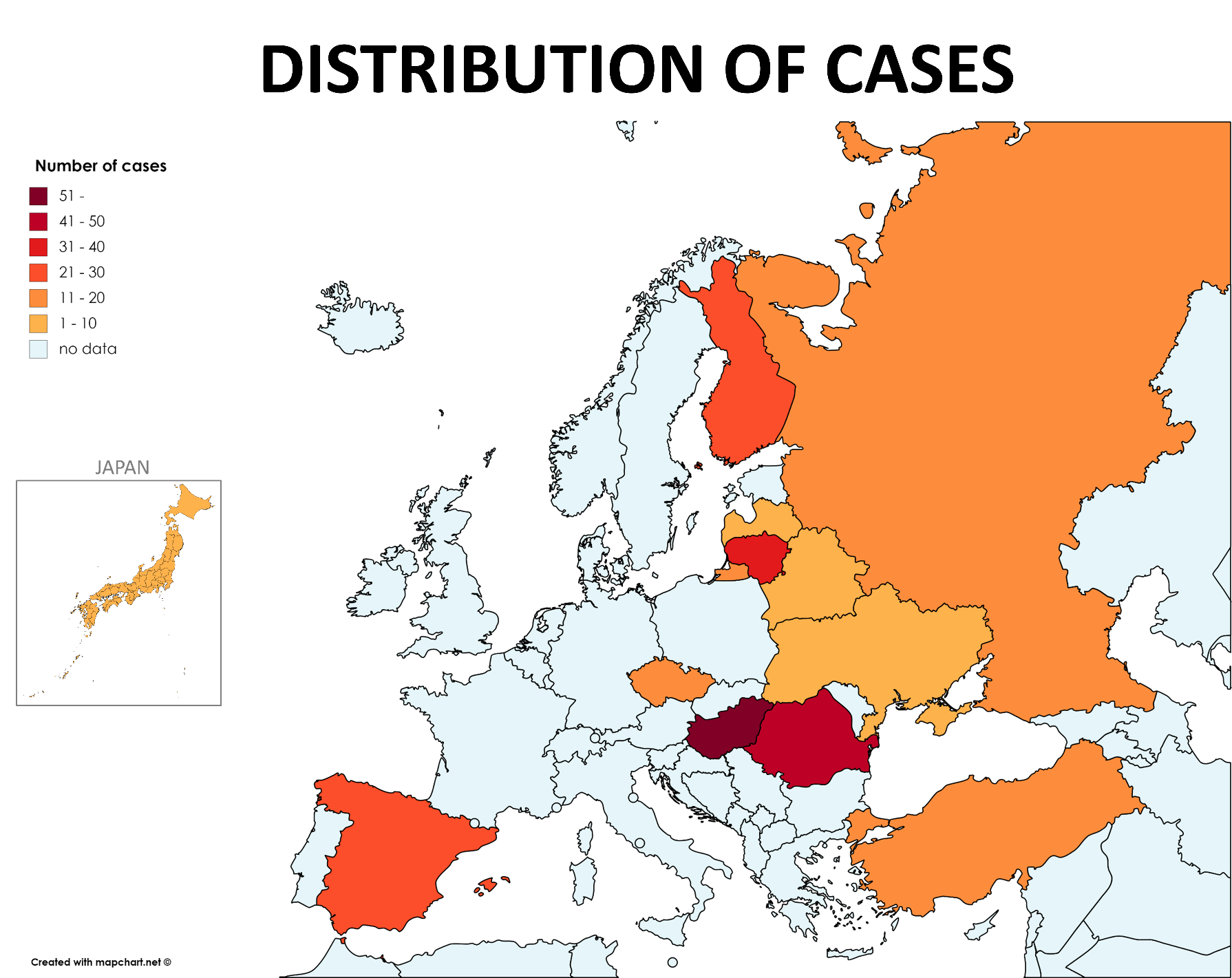
**

**Supplementary figure 1**. Countries contributing with acute pancreatitis cases to the cohort.

**Supplementary table 1**.
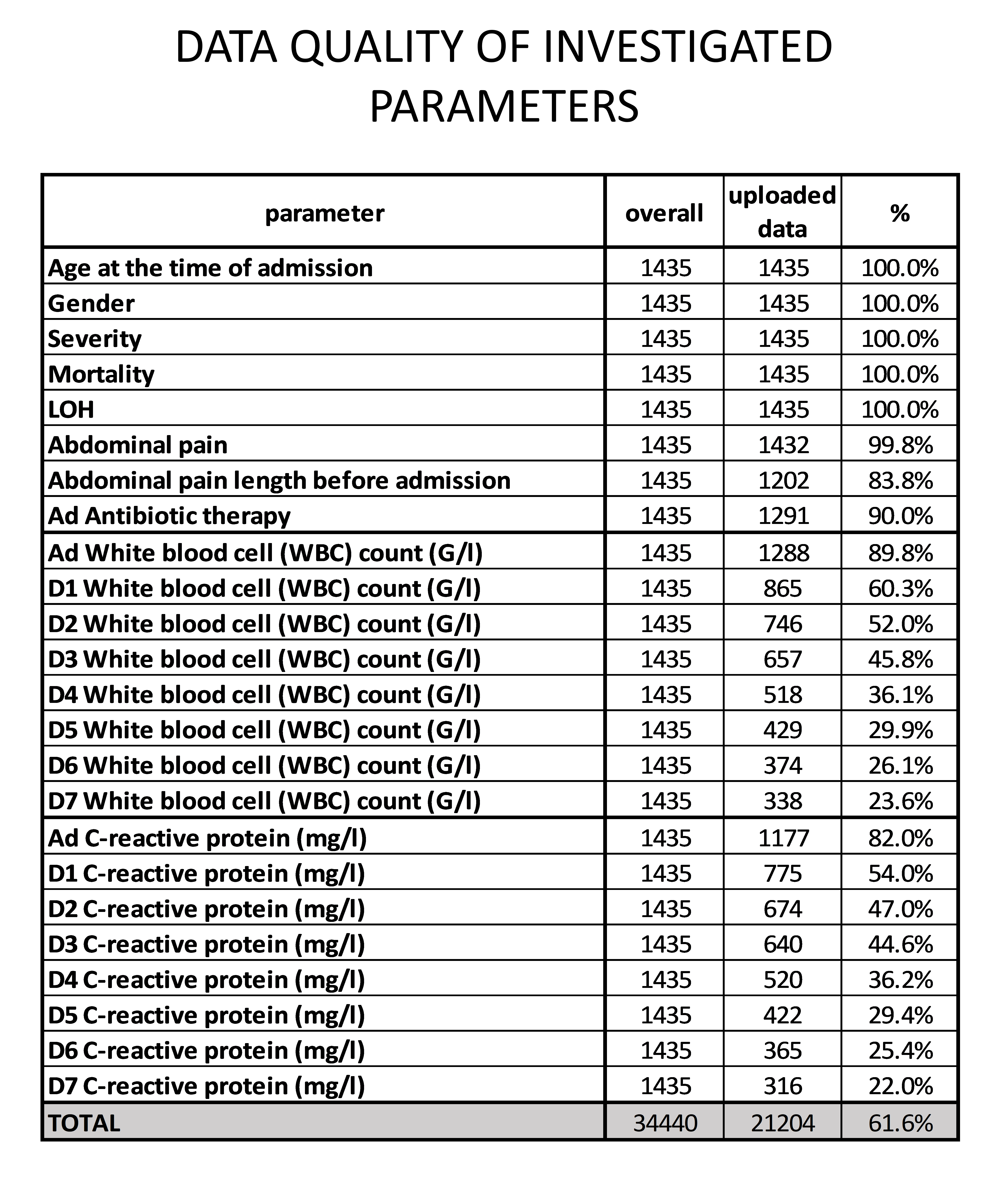
Data quality in the acute pancreatitis database.

| Study | Year | CRP cut-off (mg/l) | Timing of measurement | CRP mandatory or optional criterion? |
| --- | --- | --- | --- | --- |
| Bakker (1) | 2014 | 150 | <24 h from admission | optional |
| Besselink (2) | 2008 | 150 | <72h from symptom onset | optional |
| Dellinger (3) | 2007 | 120 | <120h from symptom onset | optional |
| Eckerwall (4) | 2006 | 150 | <48 symptom onset | mandatory |
| Hajdu (5) | 2012 | 150 | not specified exactly, but on admission | optional |
| Isenmann (6) | 2004 | 150 | <72h from symptom onset | optional |
| Kahl (7) | 2014 | 120 | not specified | mandatory |
| Kecskes (8) | 2003 | 150 | <48h from symptom onset | mandatory |
| Manes (9) | 2013 | 120 | <48h from symptom onset | mandatory |
| Maravi-Poma (10) | 2003 | 120 | not specified | optional |
| Nordback (11) | 2001 | 150 | <48h from admission | mandatory |
| Olah (12) | 2007 | 150 | <48h from symptom onset | optional |
| Petrov (13) | 2006 | 150 | <72h from symptom onset | optional |
| Piascik (14) | 2010 | 150 | <72h from symptom onset | mandatory |
| Rokke (15) | 2007 | >120 and >200 | <24h and <48h from admission | optional |
| Sadowski (16) | 2015 | 100 | not specified exactly, but on admission | optional |
| Schepers (17) | 2016 | 150 | <24h from admission | optional |
| Abraham (18) | 2013 | elevated | not specified | mandatory |
| Eatock (19) | 2005 | 150 | not specified | optional |
| NCT00894907 | 2017 | 100 | not specified | optional |
| NCT03082469 | 2017 | 100 | not specified | optional |

**Supplementary table 2.** Characteristics of CRP use as an inclusion criterion in randomized trials on acute pancreatitis.

**References for the table:**

1. Bakker OJ, van Brunschot S, van Santvoort HC, Besselink MG, Bollen TL, Boermeester MA, et al. Early versus on-demand nasoenteric tube feeding in acute pancreatitis. N Engl J Med 2014;371:1983-93.

2. Besselink MG, van Santvoort HC, Buskens E, Boermeester MA, van Goor H, Timmerman HM, et al. Probiotic prophylaxis in predicted severe acute pancreatitis: a randomised, double-blind, placebo-controlled trial. Lancet 2008;371:651-9.

3. Dellinger EP, Tellado JM, Soto NE, Ashley SW, Barie PS, Dugernier T, et al. Early antibiotic treatment for severe acute necrotizing pancreatitis: a randomized, double-blind, placebo-controlled study. Ann Surg 2007;245:674-83.

4. Eckerwall GE, Axelsson JB, Andersson RG. Early nasogastric feeding in predicted severe acute pancreatitis: A clinical, randomized study. Ann Surg 2006;244:959-65.

5. Hajdú N, Belágyi T, Issekutz A, Bartek P, Gartner B, Oláh A. Intravenous glutamine and early nasojejunal nutrition in severe acute pancreatitis -- a prospective randomized clinical study. Magy Seb 2012;65:44-51.

6. Isenmann R, Rünzi M, Kron M, Kahl S, Kraus D, Jung N, et al. Prophylactic antibiotic treatment in patients with predicted severe acute pancreatitis: a placebo-controlled, double-blind trial. Gastroenterology 2004;126:997-1004.

7. Kahl S, Schütte K, Glasbrenner B, Mayerle J, Simon P, Henniges F, et al. The effect of oral pancreatic enzyme supplementation on the course and outcome of acute pancreatitis: a randomized, double-blind parallel-group study. Jop 2014;15:165-74.

8. Kecskes G, Belagyi T, Olah A. Early jejunal nutrition with combined pre- and probiotics in acute pancreatitis--prospective, randomized, double-blind investigations. Magy Seb 2003;56:3-8.

9. Manes G, Rabitti PG, Menchise A, Riccio E, Balzano A, Uomo G. Prophylaxis with meropenem of septic complications in acute pancreatitis: a randomized, controlled trial versus imipenem. Pancreas 2003;27:e79-83.

10. Maraví-Poma E, Gener J, Alvarez-Lerma F, Olaechea P, Blanco A, Domínguez-Muñoz JE. Early antibiotic treatment (prophylaxis) of septic complications in severe acute necrotizing pancreatitis: a prospective, randomized, multicenter study comparing two regimens with imipenem-cilastatin. Intensive Care Med 2003;29:1974-80.

11. Nordback I, Sand J, Saaristo R, Paajanen H. Early treatment with antibiotics reduces the need for surgery in acute necrotizing pancreatitis--a single-center randomized study. J Gastrointest Surg 2001;5:113-8.

12. Oláh A, Belágyi T, Pótó L, Romics L Jr, Bengmark S. Synbiotic control of inflammation and infection in severe acute pancreatitis: a prospective, randomized, double blind study. Hepatogastroenterology 2007;54:590-4.

13. Petrov MS, Kukosh MV, Emelyanov NV. A randomized controlled trial of enteral versus parenteral feeding in patients with predicted severe acute pancreatitis shows a significant reduction in mortality and in infected pancreatic complications with total enteral nutrition. Dig Surg 2006;23:336-44; discussion 44-5.

14. Piaścik M, Rydzewska G, Milewski J, Olszewski S, Furmanek M, Walecki J, et al. The results of severe acute pancreatitis treatment with continuous regional arterial infusion of protease inhibitor and antibiotic: a randomized controlled study. Pancreas 2010;39:863-7.

15. Røkke O, Harbitz TB, Liljedal J, Pettersen T, Fetvedt T, Heen LØ, et al. Early treatment of severe pancreatitis with imipenem: a prospective randomized clinical trial. Scand J Gastroenterol 2007;42:771-6.

16. Sadowski SM, Andres A, Morel P, Schiffer E, Frossard JL, Platon A, et al. Epidural anesthesia improves pancreatic perfusion and decreases the severity of acute pancreatitis. World J Gastroenterol 2015;21:12448-56.

17. Schepers NJ, Bakker OJ, Besselink MG, Bollen TL, Dijkgraaf MG, van Eijck CH, et al. Early biliary decompression versus conservative treatment in acute biliary pancreatitis (APEC trial): study protocol for a randomized controlled trial. Trials 2016;17:5.

18. Abraham P, Rodriques J, Moulick N, Dharap S, Chafekar N, Verma PK, et al. Efficacy and safety of intravenous ulinastatin versus placebo along with standard supportive care in subjects with mild or severe acute pancreatitis. J Assoc Physicians India 2013;61:535-8.

19. Eatock FC, Chong P, Menezes N, Murray L, McKay CJ, Carter CR, et al. A randomized study of early nasogastric versus nasojejunal feeding in severe acute pancreatitis. Am J Gastroenterol 2005;100:432-9.

**Supplementary references**.


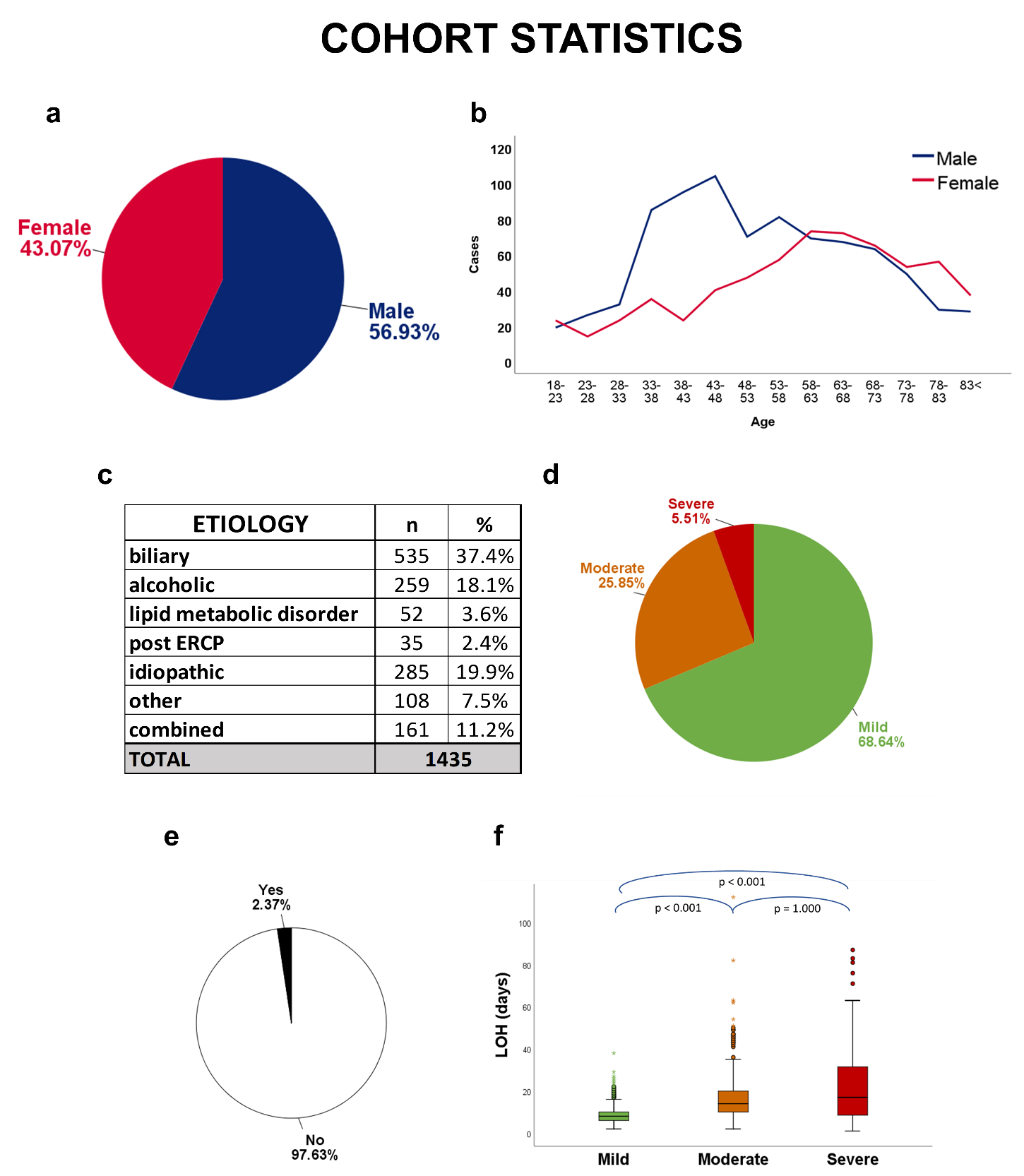


**Supplementary figure 2.** Descriptive cohort statistics, a: gender ratio, b: age distribution for genders, c: causes of acute pancreatitis, d: distribution of severity, e: mortality, f: length of hospitalization for severity grades.


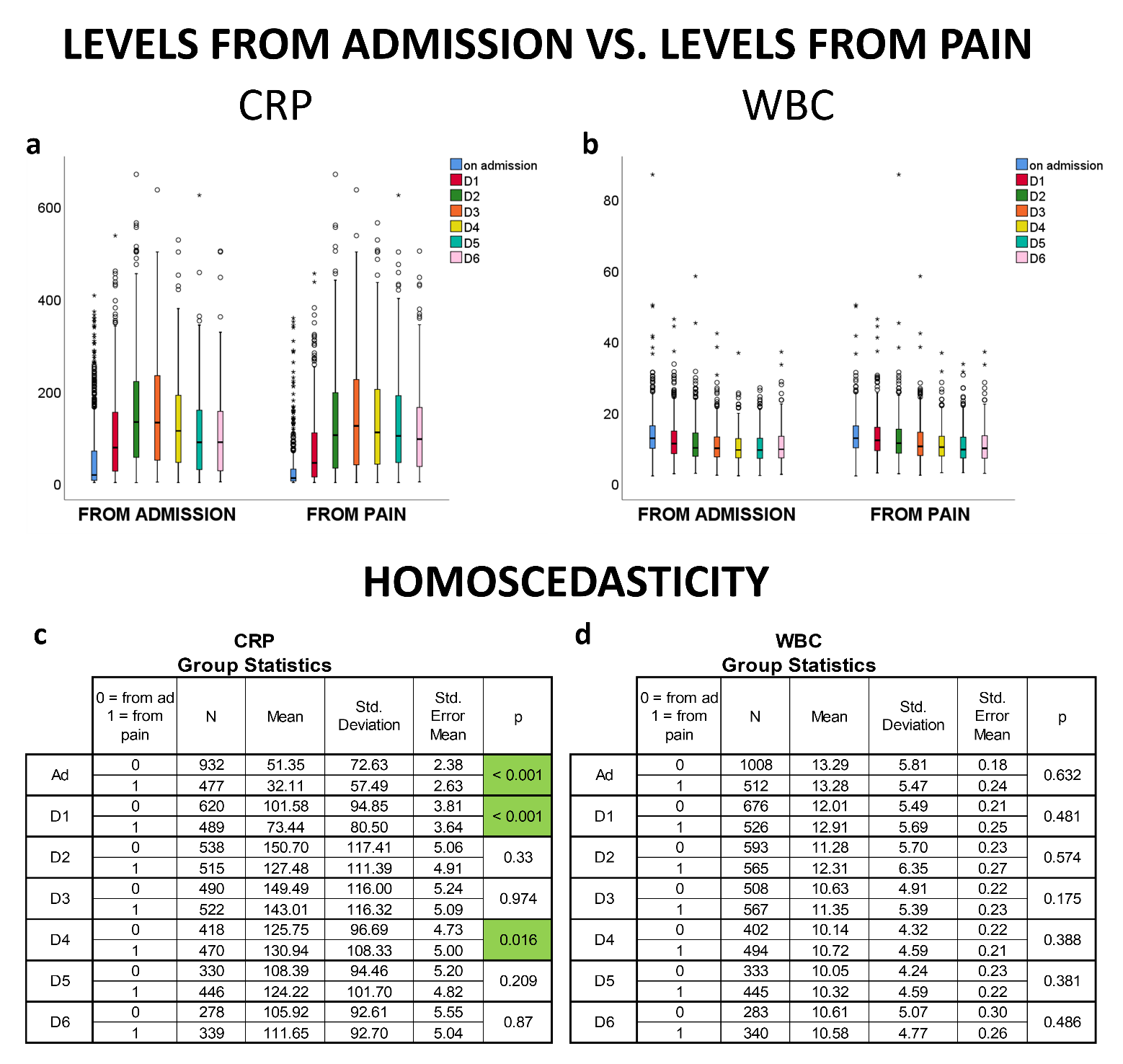
**Supplementary figure 3**. Testing of homoscedasticity of CRP (a, c) and WBC (b, d) values for the duration of symptoms.


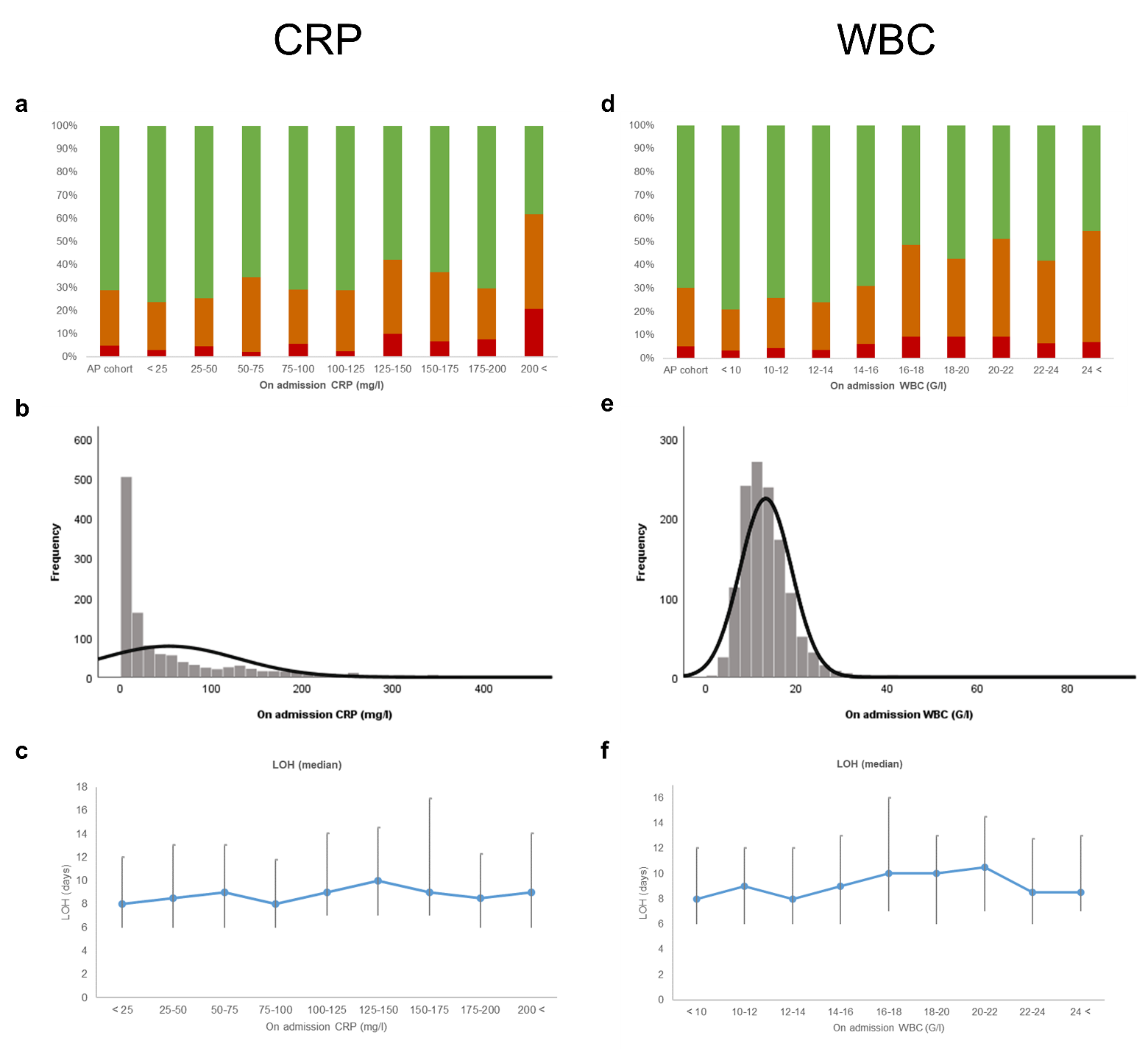
**Supplementary figure 4**.

On admission CRP: a: severity distribution for CRP categories (green: mild, amber: moderately severe, red: severe acute pancreatitis), b: number of results for CRP categories c: length of hospitalization for CRP categories.

On admission WBC: d: severity distribution for WBC categories (green: mild, amber: moderately severe, red: severe acute pancreatitis), e: number of results for WBC categories f: length of hospitalization for WBC categories.


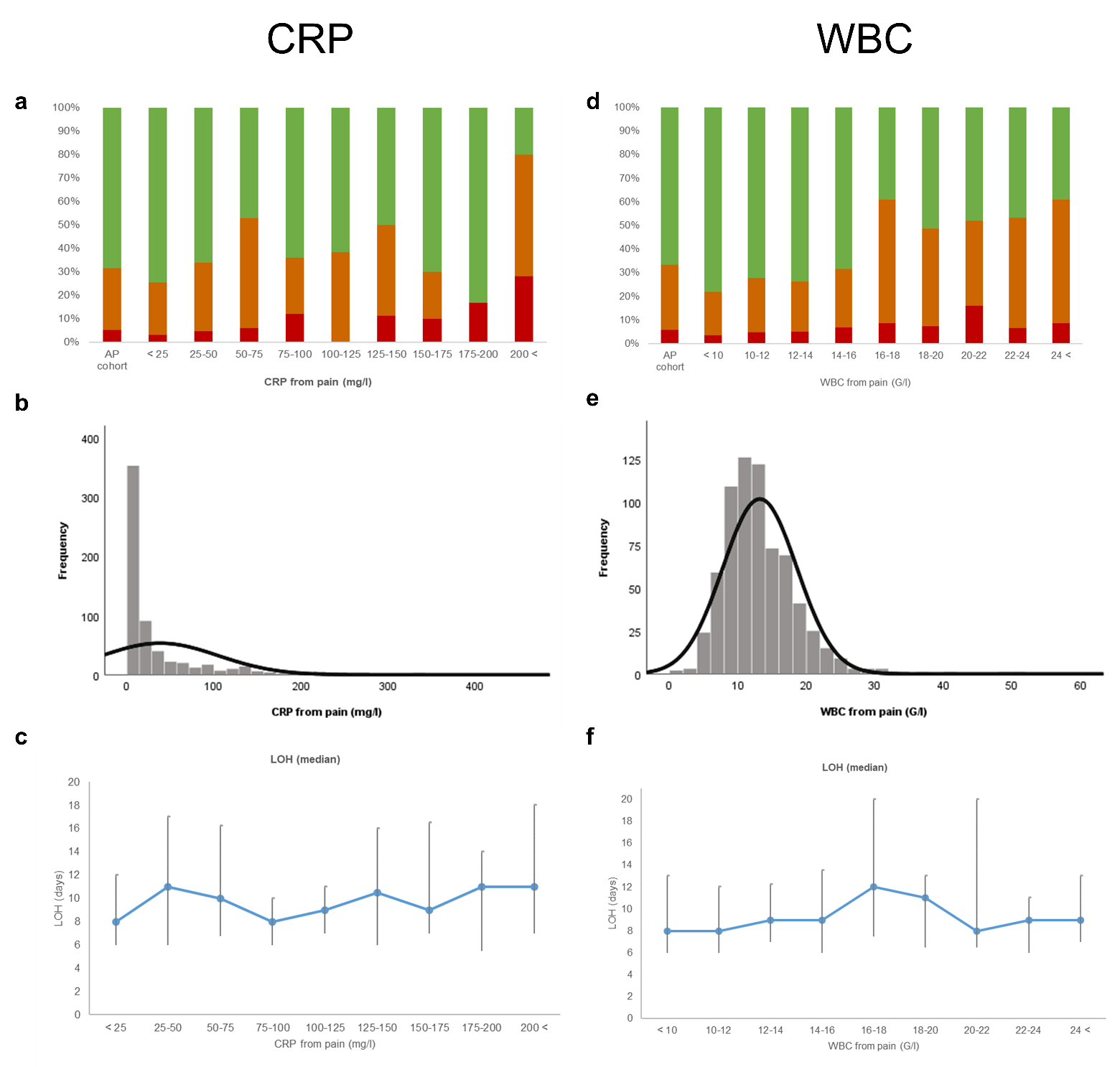
**Supplementary figure 5**.

CRP within 24 hours from the onset of pain: a: severity distribution for CRP categories (green: mild, amber: moderately severe, red: severe acute pancreatitis), b: number of results for CRP categories c: length of hospitalization for CRP categories.

WBC within 24 hours from the onset of pain: d: severity distribution for WBC categories (green: mild, amber: moderately severe, red: severe acute pancreatitis), e: number of results for WBC categories f: length of hospitalization for WBC categories.


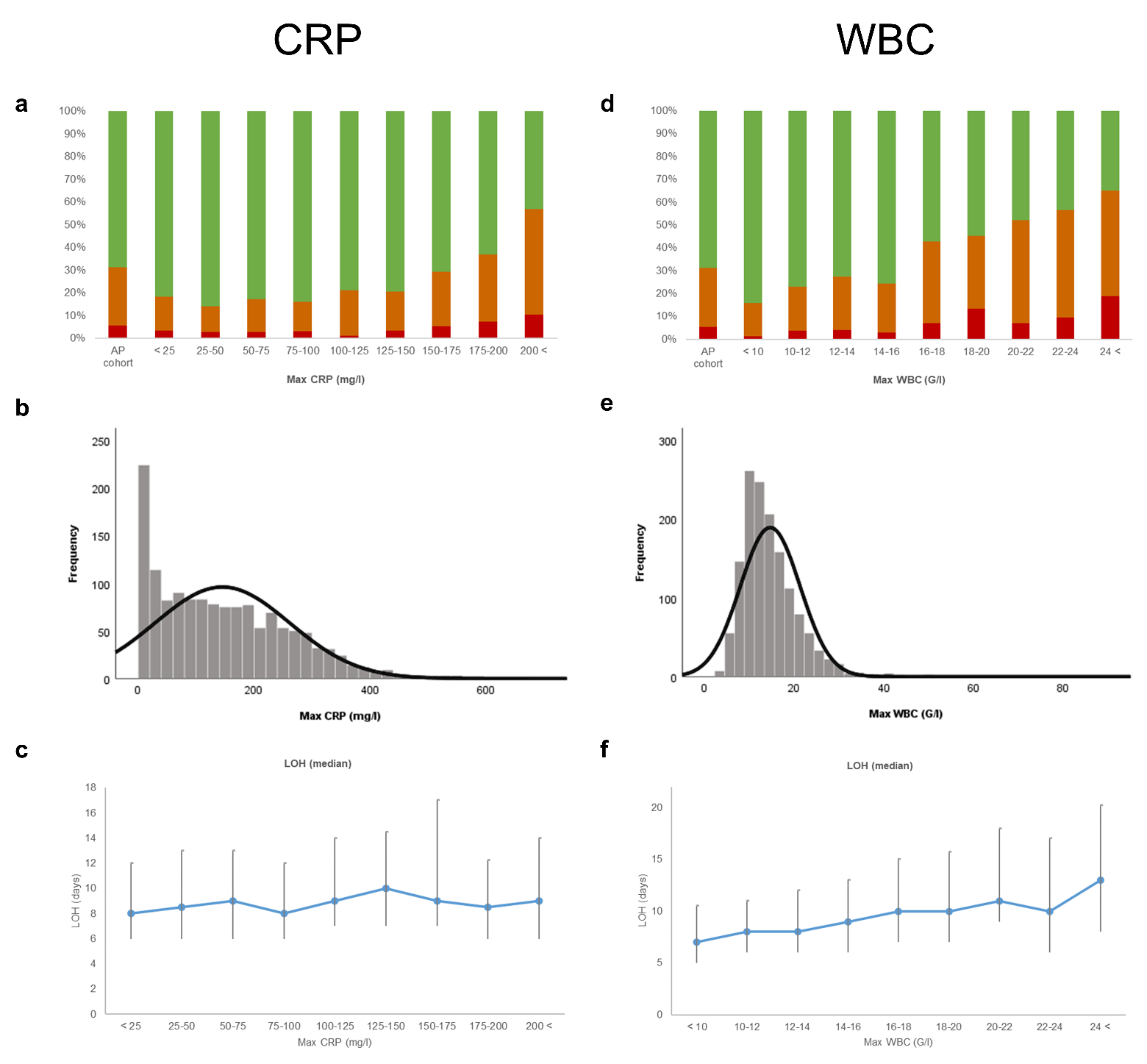
**Supplementary figure 6**.

Maximum CRP during hospitalization: a: severity distribution for CRP categories (green: mild, amber: moderately severe, red: severe acute pancreatitis), b: number of results for CRP categories c: length of hospitalization for CRP categories.

Maximum WBC during hospitalization: d: severity distribution for WBC categories (green: mild, amber: moderately severe, red: severe acute pancreatitis), e: number of results for WBC categories f: length of hospitalization for WBC categories.
